# Serum Midkine is a clinical significant biomarker for colorectal cancer and associated with poor survival

**DOI:** 10.1002/cam4.2884

**Published:** 2020-01-26

**Authors:** Marius Kemper, Wiebke Hentschel, Julia‐Kristin Graß, Bjoern‐Ole Stüben, Leonie Konczalla, Tamina Rawnaq, Tarik Ghadban, Jakob R. Izbicki, Matthias Reeh

**Affiliations:** ^1^ Department of General, Visceral and Thoracic Surgery University Medical Centre Hamburg‐Eppendorf Hamburg Germany

**Keywords:** colorectal cancer, Midkine, multimarker panel, ROC, tumormarker

## Abstract

Colorectal carcinoma (CRC) is one of the most common carcinomas worldwide. Early detection is crucial for reducing morbidity and mortality. Several promising studies described the use of midkine (MK) as a tumor marker. This study aimed to investigate a larger collective to ascertain if the preoperative serum midkine level (S‐MK) is suitable as a marker for screening and if S‐MK correlates with tumor progression and localization. It was also investigated for the first time whether patients with high S‐MK show poor survival. This prospective single‐center study included 299 patients with CRC. The preoperative serum midkine level (S‐MK) was determined using ELISA. Established tumor markers Carcinoembryonic antigen (CEA) and Carbohydrate antigen 19‐9 (CA 19‐9) were collected for comparison. The median follow‐up period was 65 months. S‐MK was significantly elevated in patients with CRC (*P* < .001). The receiver operation characteristic (ROC) curve has an area under the curve (AUC) of 0.868 (*P* < .001). A cut‐off value of 56.42 pg/mL results in a sensitivity of 84.3% and a specificity of 75.4%. In the one‐way analysis of variance (ANOVA), there were no significant correlations between S‐MK and tumor progression, localization. Furthermore, no significant correlation to CEA und CA 19‐9 could be found. Kaplan‐Meier survival analysis was able to show for the first time that patients with S‐MK of more than 225 pg/mL have a significantly shorter survival. Multivariate Cox regression showed that only CEA was an independent prognostic factor for survival. S‐MK helps estimate the prognosis for CRC and is a valuable component for developing a multimarker panel for screening and surveillance.

## Graphical abstract

This study shows that preoperative serum midkine level (S‐MK) is suitable as marker for screening of patients with colorectal carcinoma. Moreover S‐MK correlates with poor survival.

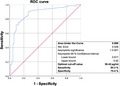

## 1 INTRODUCTION

Colorectal carcinoma (CRC) is one of the most common carcinomas worldwide.^1^ Most CRCs arise from adenomas (adenoma‐carcinoma sequence). A period of at least 10 years is assumed for the transformation of an adenoma into a carcinoma. For this reason, screening plays a major role in their prevention. CRC is seldom seen prior to the age of 40 years. In 90% of cases the disease occurs after the age of 50 years. Many guidelines recommend coloscopy as a screening tool starting at age 50.^2^, ^3^ Proof has already been presented that CRC screening reduces mortality. Despite clear consensus on this in the medical community, the percentage of patients who participate in CRC screening is unsatisfactory. Rates of participation in the United States are around 65% and in Germany 23%‐26%.^4^, ^5^ The reasons for this include the risk of bleeding and perforation, not to mention the discomfort of the exam itself. Adler et al investigated the willingness of study participants who refused a coloscopy to have a noninvasive, blood‐based screening test. A total of 97% of the patients who had rejected a coloscopy earlier were willing to take a blood test. A blood test is the type of screening test preferred by patients.^6^

Established tumor markers such as carcinoembryonic antigen (CEA) and carbohydrate antigen 19‐9 (CA 19‐9) do not have sufficient sensitivity or specificity.^7^, ^8^ However, predictive accuracy can be increased by combining these established biomarkers with new innovative ones to create a multimarker panel.^9^

Midkine (MK) is a growth factor and a promising tumor marker for different tumor entities. Physiologically MK is heavily expressed during embryogenesis. Since a low level of MK expression continues in healthy adults, a background level is to be assumed in peripheral blood.^10^ Due to the high solubility of MK in blood, the serum concentration is an approximate value for the degree of MK expression in a tumor and can be easily analyzed. Until today, there has been no uniform reference range for S‐MK since large‐scale population studies are absent.^11^ Regional divergence for S‐MK is also suspected.^12^ High expression of MK is known for numerous tumors, such as gastric cancer, esophagus squamous cell cancer, pancreatic cancer, and colorectal carcinoma.^13^, ^14^, ^15^, ^16^, ^17^, ^18^

In terms of midkine's function in tumor progression, it is known that MK contributes to neoangiogenesis and tumor cell proliferation while inhibiting apoptosis.^16^, ^19^, ^20^ In addition, MK inhibits the interaction with T cells and contributes to the expression of pro‐inflammatory cytokines such as IL‐8 and TGF‐beta. MK is also involved in the modulation of the extracellular matrix that promotes tumor cell migration.^21^ For rectal carcinoma cells Takei et al have already demonstrated that the functional loss of MK leads to a reduction in cell proliferation in vitro and a reduction in primary tumor growth in the mouse model.^16^

Krystek‐Kopracka et al have studied whether circulating serum MK (S‐MK) is suitable as a marker for CRC. The collective studied was relatively small with 105 patients. The results showed that S‐MK in the case of CRC was significantly higher compared to the control. Tumor markers are also expected to predict tumor progression. For esophagus squamous cell cancer it is known that high S‐MK prior to surgical intervention is associated with poor survival.^13^ Until today, it has not been investigated if the concentration of S‐MK in patients with CRC correlates with survival. Thus, this prospective study was to see if S‐MK can be confirmed as a tumor marker for screening in a larger collective and if a high level of S‐MK prior to surgery is associated with poor survival and is a suitable biomarker for estimating patients’ prognosis.

## 2 MATERIAL AND METHODS

### 2.1 Study population

The study included 299 patients with CRC who underwent resection with curative intent between 2002 and 2012 at the Department of General, Visceral and Thoracic Surgery at the University Medical Centre Hamburg‐Eppendorf. This patient group consisted of 108 (36%) women and 192 (64%) men. The median age was 64 years. Tumor histopathology was classified in compliance with UICC guidelines and encompassed: 55 stage I, 71 stage II, 80 stage III, and 84 stage IV. Localization of the primary tumor showed 70 ascending colon, 9 transverse colon, 5 descending colon, 51 sigmoid colon, 42 rectosigmoid junction, and 117 rectum. All included patients were treated following national guidelines.^2^ Patients with localized colon carcinoma (UICC stage I‐II) received primary resection alone. Whereas patients with advanced disease (UICC stage III‐IV) undergone adjuvant chemotherapy. Patients with advanced rectal cancer (UICC stage II‐IV) received neoadjuvant pretreatment followed by resection, unlike patients with early‐stage disease (UICC I) who were primarily operated. The median follow‐up period for the patient group was 65 months (range 61.5 to 68.5 months). The follow‐up included anamnesis, physical examination, abdominal sonography, and CEA monitoring every 6 months within the first two years and thereafter every 12 months. The first follow‐up coloscopy was within 6‐12 months and after that every 5 years. Patients with rectal cancer additionally underwent a CT scan after 3 months and sigmoidoscopy every 6 months for at least 2 years. Patients with rectal cancer received a chest x‐ray every 12 months.

Sixty‐five healthy individuals served as control. The group of healthy individuals was comprised of 43 (66%) women and 22 men (43%). The median age was 49. There were no known relevant preexisting medical conditions including no chronic inflammatory bowel disease.

The study protocol was approved by the Ethics Committee of the Medical Board in Hamburg, Germany. Written informed consent was obtained from all participants.

### 2.2 Enzyme‐Linked Immunosorbent Assay (ELISA) for S‐MK

Blood samples were taken before surgery and stored at −80°C until ELISA was performed. To determine the concentration of S‐MK in the study population, we used a commercially available MK ELISA (Antigenix America) according to the manufacturer's instructions.^22^, ^23^ Briefly described, microtiter wells precoated with anti‐human MK antibodies were incubated with the patient serum. After washing, a biotin‐labeled anti‐human MK antibody was added and the plates were incubated for 1 hour with captured MK. After thorough washing, streptavidin horseradish peroxidase conjugate was added to convert the substrate H_2_O_2_‐tetramethylbenzidine. The absorption of the solution can be analyzed photometrically at 450 nanometer (nm) (OD450) wavelengths in the microplate reader (Dynatech MR 500). To measure the exact concentration of the analytes in the liquid phase, a calibration curve was plotted based on samples with known concentrations. MK concentration is given in pg/mL.

### 2.3 Carcinoembryonic antigen and CA 19‐9 assay

CEA and CA 19‐9 were routinely determined by Electro‐chemiluminescence immunoassay (ECLIA) following the manufacturer's instructions (Roche Diagnostics Deutschland GmbH, Mannheim, Germany). The cut‐off value of CEA and CA 19‐9 were 3.4 µg/L and 27 kU/L, respectively.

### 2.4 Statistical analysis

The Statistical Package for Social Sciences (SPSS^®^) for Mac (Version 25) (IBM) was used for the statistical analysis. Descriptive statistics were used to describe patient baseline characteristics. Comparisons between unpaired groups were made using the Mann‐Whitney U test. Correlations between parameters were performed using one‐way analysis of variance (ANOVA). To ascertain the suitability of S‐MK as tumor marker, the receiver operating characteristic (ROC) curve was determined and the resulting area under the curve (AUC) calculated. The accuracy of the markers was evaluated depending on the AUC: 0.5‐0.59 = fail; 0.6‐0.69 = poor; 0.7‐0.79 = fair; 0.8‐0.89 = good; 0.9‐1 = excellent. Using the ROC curve, an optimized cut‐off value was defined for S‐MK in regard to sensitivity and specificity. Events considered for survival analysis were death due to cancer diagnosis. When no event was recorded, the patients were censored at the last contact for statistical evaluation. Survival curves for the overall survival of the patients were plotted (Kaplan‐Meier method) and analyzed by implementing the log‐rank test. The overall survival (OS) was computed as the time period from the date of surgery to either the date of death or last follow‐up, whichever occurred first. Cox regression analysis was performed for multivariate analysis. Statements of significance refer to *P*‐values of two‐tailed tests that were less than .05.

## 3 RESULTS

**Figure 1:**
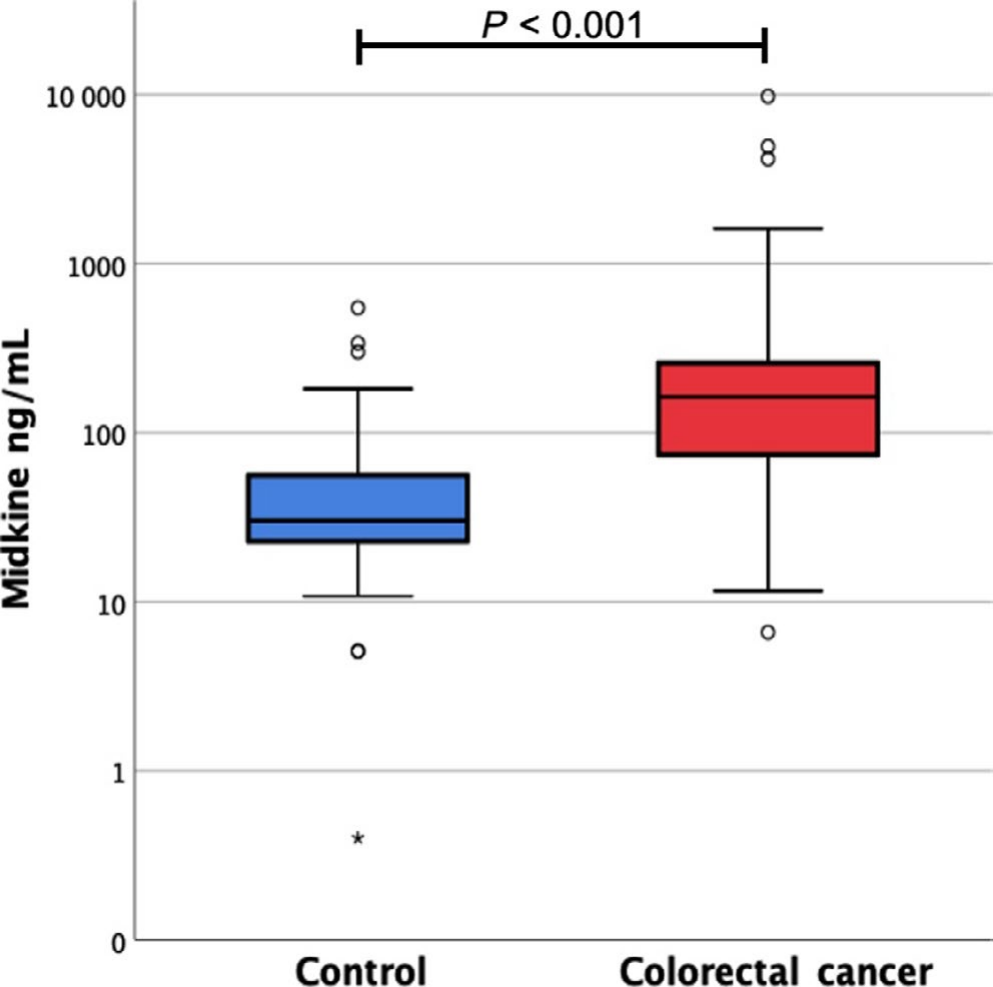
The midkine concentration plotted here logarithmically is significantly elevated in the preoperative blood serum of patients with colorectal carcinoma (red) compared to the control subjects (blue) with *P* < .001

We compared the concentration of S‐MK in patients with CRC and healthy control subjects. The mean S‐MK concentration for patients with CRC was significantly higher at 257 pg/mL compared to healthy control subjects for whom a mean of 55 pg/mL was measured (*P* < .001, Table 1, Figure 1).

**Table 1:** Midkine (pg/mL) control vs colorectal carcinoma

	N	Midkine pg/mL Mean	Midkine pg/mL Std. Error Mean	*P*‐value
Control	65	55	11	**<.001**
Colorectal carcinoma	299	257	39	

**Figure 2:**
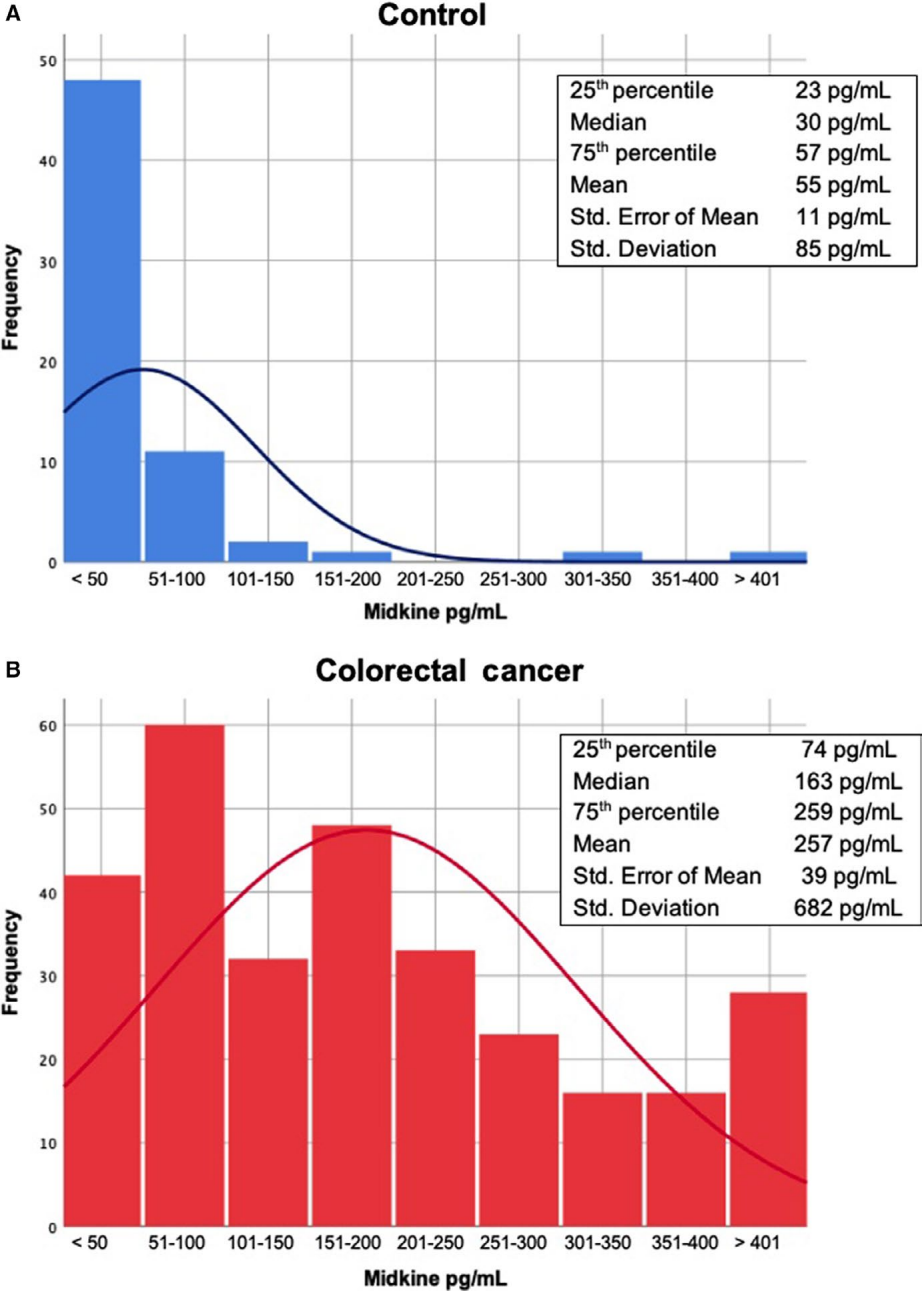
Histogram and distribution curve of the grouped preoperative midkine concentration of the control subjects (A, blue) and the patients with colorectal carcinoma (B, red)

**Figure 3:**
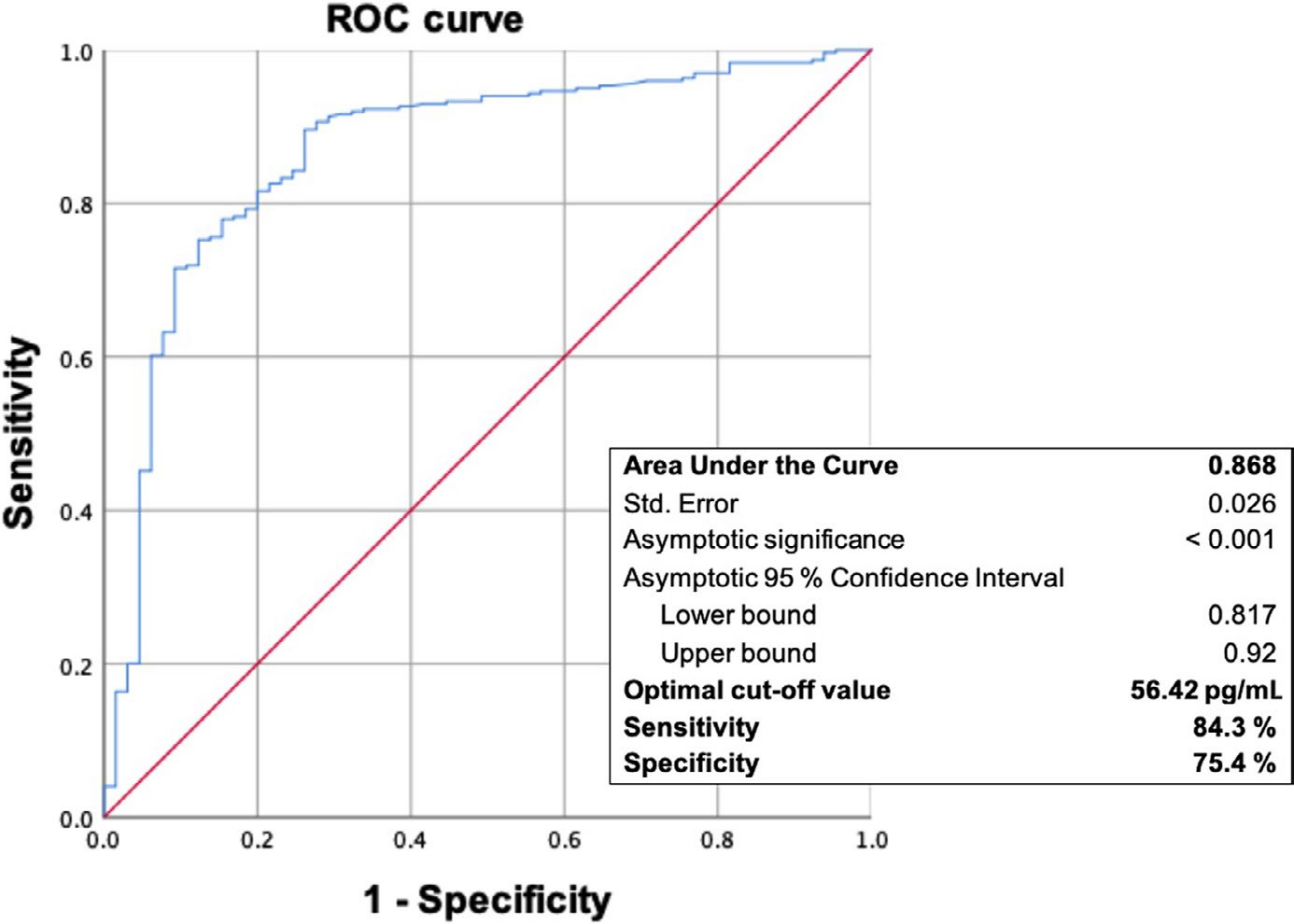
Receiver operating characteristic curve and diagnostic values for midkine as tumor marker for colorectal carcinoma. The area under the curve is 0.868 indicating that the diagnostic accuracy can be described as good. The optimal cut‐off value of 56.42 pg/mL yields a balanced relationship between 84.3% sensitivity and 75.4% specificity

The frequency distribution of S‐MK in patients with CRC and the control group is presented as a histogram with a distribution curve in Figure 2. To analyze the diagnostic power of S‐MK as tumor marker for CRC, a receiver operation characteristic (ROC) curve was mapped out. The resulting area under the curve (AUC) was 0.868 (*P* < .001). Hence, the diagnostic accuracy can be described as good. There is a balanced relationship between sensitivity (84.3%) and specificity (75.4%) when the cut‐off value is set at 56.42 pg/mL (Figure 3).

One‐way analysis of variance (ANOVA) was used to investigate if S‐MK correlates with disease progression, precise tumor stage, lymph node metastasis, distant metastasis, resection status, grading, UICC classification, tumor localization, CEA, and CA 19‐9. For S‐MK no significant correlations could be detected (Table 2). However, two trends for S‐MK can be described involving grading and tumor localization. High‐grade tumors with a mean of 405 pg/mL tend to have a higher S‐MK in comparison to low‐grade tumors with a mean of 231 pg/mL. Likewise, there is a nonsignificant trend visible for tumor localization. Tumors of the sigmoid colon (270 pg/mL), rectosigmoid junction (309 pg/mL), and rectum (286 pg/mL) tend to show a higher S‐MK than tumors of the ascending (193 pg/mL), transverse (159 pg/mL), and descending colon (151 pg/mL).

**Table 2:** Association of Midkine with CRC progression, location, CEA, and CA 19‐9

	Number of patients	Midkine (pg/mL) Mean (95%CI)	ANOVA *P*‐value
Gender			.162
Female	108	183 (157‐210)	
Male	191	298 (178‐419)	
Tumor stage			.725
pT1	26	199 (142‐256)	
pT2	46	210 (165‐255)	
pT3	166	308 (171‐446)	
pT4	51	184 (119‐249)	
Lymph node metastasis			.848
pN0	152	257 (128 ‐ 384)	
pN1	64	221 (164‐278)	
pN2	76	288 (123‐452)	
Distant metastasis			.493
M0	59	360 (30‐690)	
M1	89	256 (141‐371)	
Residual tumor			.89
R0	169	193 (133‐252)	
R1	12	148 (75‐220)	
R2	1	86,40	
Grading			.117
Low grade	229	231 (145‐316)	
High grade	49	405 (145‐664)	
UICC classification			.718
I	55	209 (167‐250)	
II	71	336 (63‐610)	
III	80	226 (123‐330)	
IV	84	261 (140‐383)	
Tumor location			.82
Ascending colon	70	193 (141‐244)	
Transverse colon	9	159 (55‐262)	
Descending colon	5	151 (9,25‐293)	
Sigmoid colon	51	270 (102‐440)	
Rectosigmoid junction	42	309 (76‐543)	
Rectum	117	286 (122‐450)	
CEA			.453
<3.4 µg/L	74	234 (115‐352)	
≥3.4 µg/L	77	350 (70‐630)	
CA 19‐9			.312
<27 kU/L	85	350 (103‐598)	
≥27 kU/L	51	244 (53‐436)	

**Figure 4:**
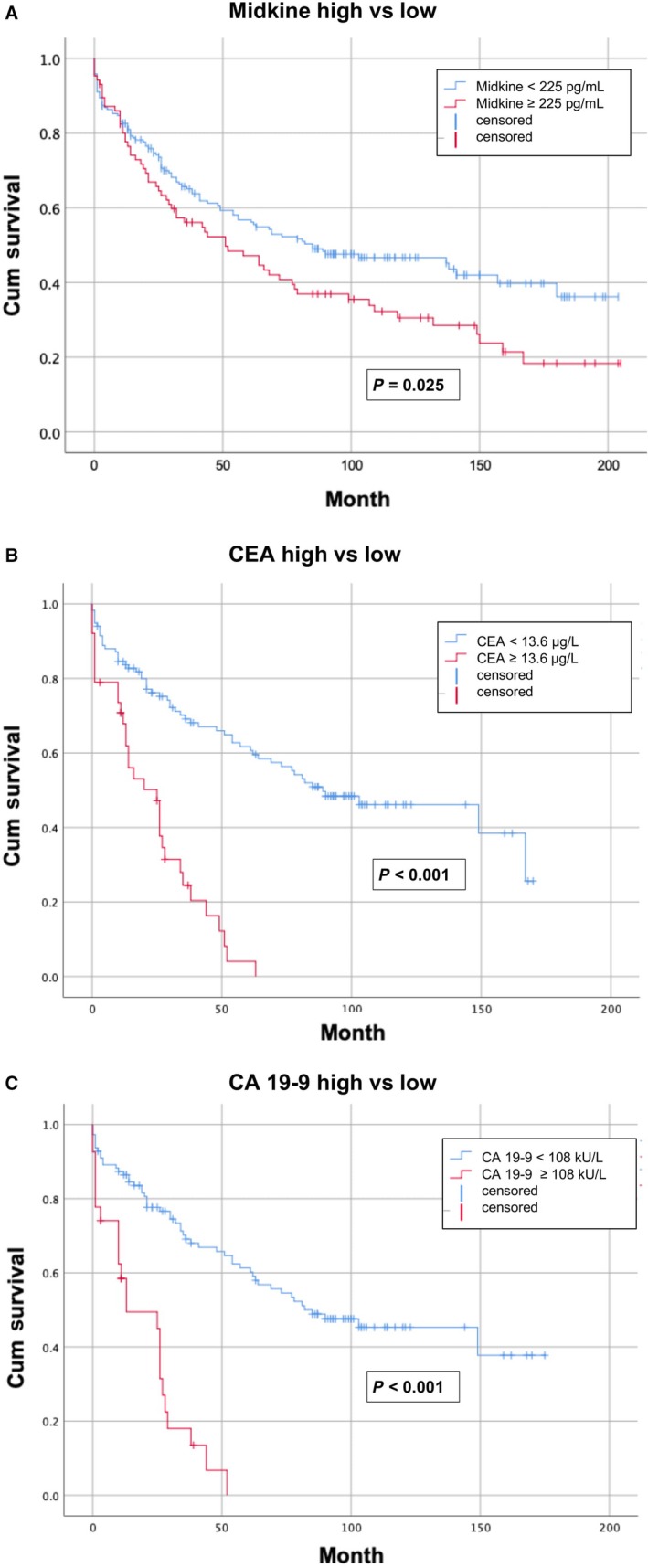
Kaplan‐Meier survival curves for 299 patients with colorectal carcinoma. The group with a comparably low serum midkine concentration < 225 pg/mL (low S‐MK group) has a significantly better cumulative survival compared to the group with a high expression of midkine (high S‐MK group) (A, *P* = .025). Patients of the low CEA (B) and CA 19‐9 group (C) also showed significantly favorable survival rates (*P* < .001)

To analyze the relation of S‐MK and, for comparison purposes, CEA and CA 19‐9 with survival Kaplan‐Meier analysis were performed. The median follow‐up period was 65 months. To achieve sufficient discriminatory power, the cut‐off value for S‐MK before optimized in the ROC analysis was quadrupled. Likewise, the cut‐off values of CEA and CA 19‐9 were quadrupled for survival analysis. Accordingly, patients with S‐MK less than 225 pg/mL were assigned to the low S‐MK group. This corresponded to 69% of the patients. The remaining patients were assigned to the high S‐MK group. The Kaplan‐Meier survival analysis showed a significantly higher median survival of 85 months for patients in the low S‐MK group compared to 53 months for the high S‐MK group (*P* = .025, Figure 4A). The 5‐year survival rate for the low S‐MK group was 53% and 42% for the high S‐MK group. Patients in the low CEA and CA 19‐9 group also showed significantly favorable survival rates (*P* < .001, Figure 4B,C).

Survival of patients from the early (2002‐2006) and late study period (2007‐2012) does not show a significant difference (*P* = .89, data not shown). To examine whether S‐MK depends on adjuvant or neoadjuvant treatment therapy, adjusted subgroup analysis was performed. The difference in survival between low and high S‐MK groups tended to be higher within the adjuvant or neoadjuvant treatment subgroup. Due to the low number of patients in the subgroup, this effect was not statistically significant (data not shown).

Multivariate Cox Regression showed that only CEA > 13.6 4 µg/L was an independent prognostic factor for survival (Hazard Ratio = 2.55, *P* = .014, Table 3).

**Table 3:** Univariate and multivariate analysis of risk factors for survival

	Log rank	Cox regression		
	*P*‐value	Hazard ratio	95% CI	*P*‐value
Gender	.112			
Female		1		
Male		1.61	0.95‐1.62	.080
Age	**<.001**			
<65		1		
≥65		1.65	0.98 ‐ 1.65	.058
UICC classification	**<.001**			
I		1		
II		0.76	0.31 ‐ 1.84	.539
III		0.61	0.26 ‐ 1.43	.605
IV		1.13	0.49 ‐ 2.63	.770
Serum Midkine Level	**.025**			
<225 pg/mL		1		
≥225 pg/mL		1.28	0.75 ‐ 2.19	.35
CEA	**<.001**			
<13.6 µg/L		1		
≥13.6 µg/L		2.55	1.21‐5.37	**.014**
CA‐19.9	**<.001**			
<108 kU/L		1		
≥108 kU/L		1.804	0.84‐3.87	.130

## 4 DISCUSSION

In the literature, great potential is ascribed to S‐MK for screening patients with CRC.^14^, ^24^, ^25^, ^26^, ^27^, ^28^ Krystek‐Kopracka et al analyzed a smaller patient group to see if circulating MK in serum (S‐MK) is suitable as a tumor marker for CRC. It was found that S‐MK in CRC patients was significantly higher than in the control group. Neither Krystek‐Kopracka et al nor others have investigated to date, if preoperative S‐MK correlates with survival of CRC patients and, as a result, is suitable for estimating prognosis. This prospective single‐center study aimed to investigate, on the one hand, if S‐MK can be confirmed as a diagnostic marker in a larger collective and on the other if S‐MK enables an estimation of the prognosis in comparison with the established tumor markers CEA and CA 19‐9.

To do this, S‐MK in blood samples taken from 299 patients prior to surgery was analyzed using ELISA. There is currently no established normal range for S‐MK since large‐scale population studies do not exist.^11^ Regional divergence is also suspected for S‐MK.^12^ For this reason, we measured S‐MK in healthy test subjects in a methodically identical manner. S‐MK was significantly higher in patients with CRC compared to the control subjects (*P* < .001). This finding in a larger collective is able to confirm the results of previous studies.^14^, ^24^, ^25^, ^26^, ^27^, ^28^

Furthermore, we tested the ability of midkine to differentiate between CRC patients and healthy subjects. A receiver operation characteristic (ROC) curve was calculated for S‐MK for which an area under the curve (AUC) of 0.868 was determined. This indicates that the diagnostic accuracy is to be interpreted as good. Assuming a cut‐off value of 56.42 pg/mL, it was possible to achieve a balanced relationship between sensitivity (84.3%) and specificity (75.4%). Krystek‐Kopracka et al were able to show in a direct comparison that S‐MK is superior to CEA regarding sensitivity. In national guidelines, the determination of CEA continues to be recommended for detecting CRC relapse.^2^, ^29^ It is known that S‐MK decreases after tumor resection.^30^ Hence, S‐MK appears suitable to detect relapses in posttherapy monitoring.

One‐way analysis of variance (ANOVA) was performed to evaluate if S‐MK correlates with disease progression. No significant correlations could be determined regarding tumor stage (*P* = .725), lymph node metastasis (*P* = .848), distant metastasis (*P* = .493), resection status (*P* = .89), or UICC classification (*P* = .718). High‐grade tumors tend to have a higher S‐MK compared to low‐grade tumors (*P* = .117). It is interesting to note that the concentration of midkine does not increase with primary tumor stage and UICC classification, but does tend to be elevated in the case of high‐grade tumors. It can be hypothesized that midkine is primarily relevant for carcinogenesis and plays a minor role in tumor progression. An association between S‐MK and lymph node metastasis, as described previously by Song et al, cannot be confirmed.^31^ In addition, there is a nonsignificant trend involving tumor localization (*P* = .82). Tumors of the sigmoid colon (270 pg/mL), rectosigmoid junction (309 pg/mL), and rectum (286 pg/mL) tend to show a higher S‐MK than tumors of the ascending (193 pg/mL), transverse (159 pg/mL), and descending colon (151 pg/mL), as already observed by Krystek‐Kopracka et al^12^ CEA and CA 19‐9 were not associated with S‐MK. Therefore it seems reasonable to assume that the combination of S‐MK with CEA and CA 19‐9 could increase the sensitivity.

For the first time, it was possible to demonstrate that patients with S‐MK higher than 225 pg/mL have a significantly shorter survival (*P* = .025, Log‐rank test). Likewise, the 5‐year survival rate in the high S‐MK group was lower with 42% than the survival rate in the low S‐MK group for which the rate was 53%. No significant difference has been found concerning the survival of patients of the early and late cohorts. It can therefore be concluded the overall progress in the treatment of CRC is not reflected in our survival data. Patients of the high CEA and CA 19‐9 group showed significantly reduced survival. Multivariate Cox Regression showed that CEA > 13.6 4 µg/L was an independent prognostic factor for survival. The HR of S‐MK was 1.28. This trend did not reach statistical significance in multivariate analysis.

In summary, S‐MK can be confirmed as a good tumor marker for CRC. As well as CEA and CA 19‐9, high preoperative S‐MK correlates with poorer survival in univariate analysis and is thus helpful for estimating prognosis. An explanation is posited in the study published by Takei et al in which it was shown that the functional loss of midkine in rectal carcinoma cells leads to a reduction in cell proliferation in vitro and of primary tumor growth in the mouse model.^16^ An association between S‐MK and survival was also shown for patients with neuroblastoma and esophageal cancer.^13^, ^32^ A recent study of Ito et al evaluated the diagnostic impact of S‐MK in patients with gastric adenocarcinoma. Briefly, patients with low S‐MK levels tended to have a favorable prognosis. The difference was not significant due to the low number of included patients.^18^

Since midkine can also be increased in the case of other tumor entities, the use of midkine would be most meaningful as part of a multimarker panel^9^, ^33^, ^34^ for CRC screening and surveillance. A reliable multimarker panel can in future be an attractive alternative to the unpopular coloscopy. The expected better level of compliance than is seen now for coloscopy can lead to a decrease in morbidity and mortality as a result of early detection. The molecular biological function of midkine in CRC is not completely understood at this time. For this reason, new in vitro and in vivo studies are needed.

## CONFLICT OF INTEREST

The authors have no conflict of interest.

## Data Availability

The data that support the findings of this study are available from the corresponding author upon reasonable request.
